# Genetic and neural mechanisms of sleep disorders in children with autism spectrum disorder: a review

**DOI:** 10.3389/fpsyt.2023.1079683

**Published:** 2023-05-02

**Authors:** Qi Ji, Si-Jia Li, Jun-Bo Zhao, Yun Xiong, Xiao-Hui Du, Chun-Xiang Wang, Li-Ming Lu, Jing-Yao Tan, Zhi-Ru Zhu

**Affiliations:** ^1^Department of Psychology, Army Medical University, Chongqing, China; ^2^College of Basic Medicine, Army Medical University, Chongqing, China; ^3^College of Educational Sciences, Chongqing Normal University, Chongqing, China

**Keywords:** sleep disorders, autism spectrum disorder, neural mechanism, wakening time, NREM sleep, REM sleep, sleep–wake rhythm, genetic mechanism

## Abstract

**Background:**

The incidence of sleep disorders in children with autism spectrum disorder (ASD) is very high. Sleep disorders can exacerbate the development of ASD and impose a heavy burden on families and society. The pathological mechanism of sleep disorders in autism is complex, but gene mutations and neural abnormalities may be involved.

**Methods:**

In this review, we examined literature addressing the genetic and neural mechanisms of sleep disorders in children with ASD. The databases PubMed and Scopus were searched for eligible studies published between 2013 and 2023.

**Results:**

Prolonged awakenings of children with ASD may be caused by the following processes. Mutations in the *MECP2, VGAT* and *SLC6A1* genes can decrease GABA inhibition on neurons in the locus coeruleus, leading to hyperactivity of noradrenergic neurons and prolonged awakenings in children with ASD. Mutations in the *HRH1, HRH2*, and *HRH3* genes heighten the expression of histamine receptors in the posterior hypothalamus, potentially intensifying histamine’s ability to promote arousal. Mutations in the *KCNQ3* and *PCDH10* genes cause atypical modulation of amygdala impact on orexinergic neurons, potentially causing hyperexcitability of the hypothalamic orexin system. Mutations in the *AHI1*, *ARHGEF10*, *UBE3A*, and *SLC6A3* genes affect dopamine synthesis, catabolism, and reuptake processes, which can elevate dopamine concentrations in the midbrain. Secondly, non-rapid eye movement sleep disorder is closely related to the lack of butyric acid, iron deficiency and dysfunction of the thalamic reticular nucleus induced by *PTCHD1* gene alterations. Thirdly, mutations in the *HTR2A, SLC6A4*, *MAOA, MAOB*, *TPH2*, *VMATs*, *SHANK3,* and *CADPS2* genes induce structural and functional abnormalities of the dorsal raphe nucleus (DRN) and amygdala, which may disturb REM sleep. In addition, the decrease in melatonin levels caused by *ASMT*, *MTNR1A*, and *MTNR1B* gene mutations, along with functional abnormalities of basal forebrain cholinergic neurons, may lead to abnormal sleep–wake rhythm transitions.

**Conclusion:**

Our review revealed that the functional and structural abnormalities of sleep–wake related neural circuits induced by gene mutations are strongly correlated with sleep disorders in children with ASD. Exploring the neural mechanisms of sleep disorders and the underlying genetic pathology in children with ASD is significant for further studies of therapy.

## 1. Introduction

According to the fifth edition of the Diagnostic and Statistical Manual of Mental Disorders (DSM-5), autism spectrum disorder (ASD) is a neurodevelopmental disorder characterized by social communication deficits, restricted interests, and repetitive behaviors. Approximately 1/100 children are diagnosed with ASD around the world, and the prevalence has been increasing over time (1).

Sleep disorders are common clinical symptoms in children with ASD, with a rate of nearly 50% (2). Clinical evidence has shown that sleep disorders can exacerbate ASD symptomatology (3–6). Firstly, sleep disorders are closely related to the impairment of social ability in children with ASD. Children with ASD with sleep disorders have further diminished social functioning and lower quality of life than those without sleep disorders (7). Sleep problems in autistic adolescents may lead to difficulties regulating social interactions and cause disharmonious relationships with peers (8). Secondly, sleep disorders can aggravate repetitive behaviors in children with ASD. The parental questionnaire revealed a significant correlation between Children’s Sleep Habits Questionnaire scores and Repetitive Behavioral Questionnaire-2 scores (9). Children with ASD with poor sleep quality have more repetitive behaviors (10, 11). Thirdly, sleep disorders may be a mediator of cognitive function deficits in children with ASD. Sleep is important for many complex physiological processes, such as cognitive development, learning and memory processes (12, 13). Sleep disorders can exacerbate the impairment of memory consolidation in children with ASD (5). Children with ASD who slept longer performed better on working memory tests and had higher rates of correct hits in the attention task. There was a linear relationship between poor working memory and sleep disorders in children with ASD (14). Children with ASD showed worse narrative abilities than their healthy peers (15). In addition, sleep disorders can also affect the language function of children with ASD (4). At the same time, sleep disorders of children with ASD increase the difficulty and cost of care, placing a heavy burden on family and society (16).

The main types of sleep disorders in children with ASD are difficulties in falling asleep and maintaining sleep, which manifest as long sleep latency, nighttime waking, and reduced sleep efficiency (17). Compared to healthy controls, children with ASD experience a 30–45 min prolongation of sleep latency on weekdays, generally lower sleep efficiency, and an average nighttime waking time of 2–3 h (17). Healthy individuals’ sleep–wake cycle is divided into three parts based on the electroencephalogram (EEG) and behavioral characteristics: the awakening period, non-rapid eye movement (NREM) sleep, and rapid eye movement (REM) sleep (18). Characteristics of NREM sleep include sharp-wave ripples, cortical slow oscillations, delta waves, and spindles. REM sleep is associated with theta oscillations (19, 20). Excess synapses are removed during NREM sleep and hippocampal neural activity during REM sleep is critically involved in memory consolidation (19, 20). Polysomnography analysis showed high rates of EEG abnormalities during sleep in children with ASD. Paroxysmal slowing and epileptiform abnormalities in EEG recordings were found in children with ASD with or without a history of seizures (21). Abnormalities of NREM sleep include a reduction in NREM sleep duration, K-complex density, and density of spindle activity. In addition, children with ASD also exhibit shorter REM sleep duration, prolonged REM latency, increased theta activity during REM sleep, and circadian rhythm sleep–wake disorders (22). The pathological mechanisms of sleep disorders in children with autism are still unclear. The lack of discussion on genetic and neural mechanisms has hindered the exploration of clinical treatment.

The purpose of this review is to summarize the genetic and neural mechanisms of sleep disorders in children with ASD from 2013 until now. We searched the databases of PubMed and Scopus for eligible studies and limited our search to publications between 2013 and 2023. We included articles that were peer-reviewed, written in English, purposefully addressed genetic and neural mechanisms, focused on sleep disorders, and included autism. Articles were excluded if they were not written in English, were non-peer reviewed, did not primarily focus on individuals with both autism and sleep disorders, did not clearly address genetic and neural mechanisms, or were abstracts, dissertations, methodological papers, or conference papers. The search strategy identified 397 documents, which were reduced to 65 included articles after applying exclusion criteria.

## 2. Genetic and neural mechanisms of the awakening time abnormalities in children with ASD

### 2.1. Locus Coeruleus and the noradrenergic system

**Figure 1:**
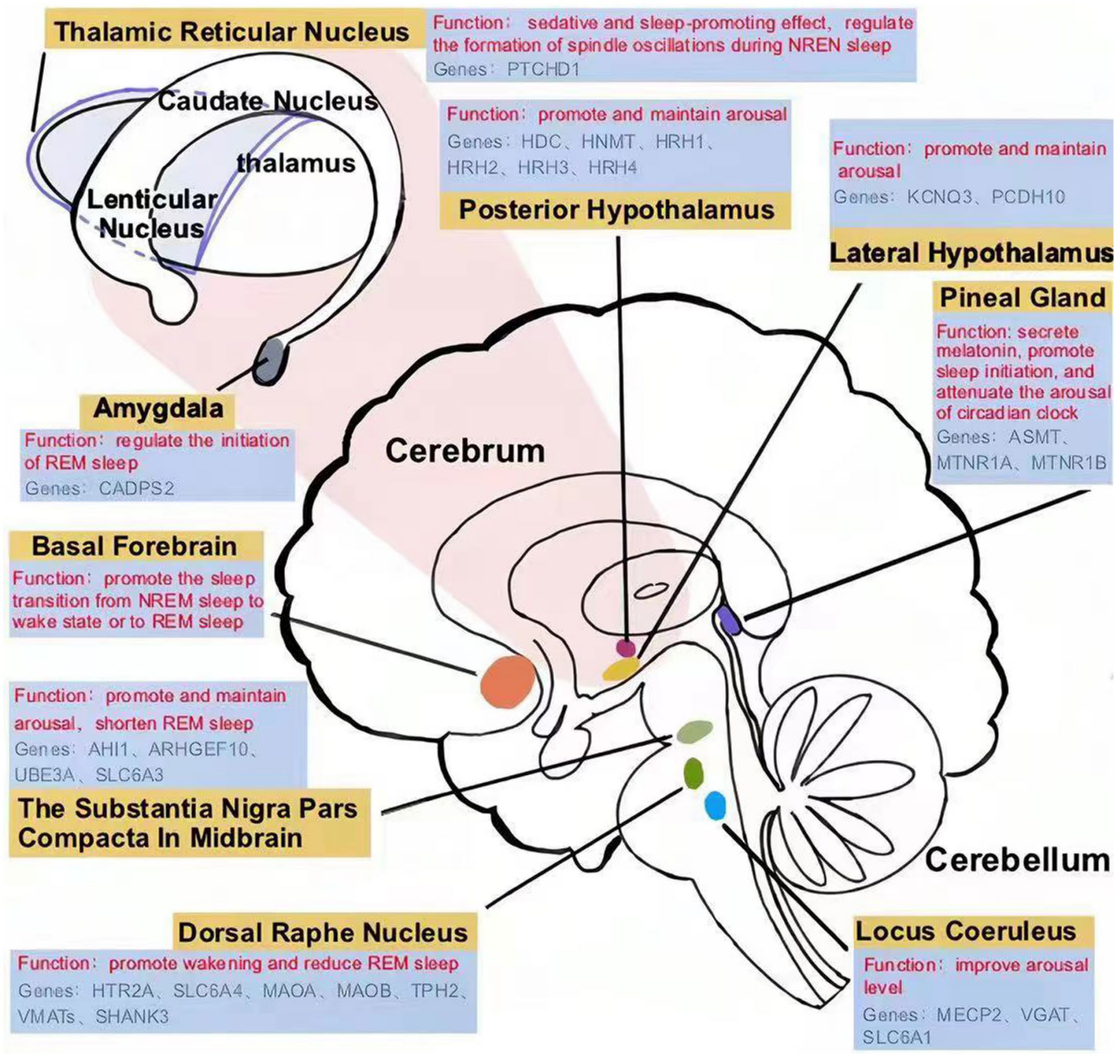
Sleep disorder-related nucleus and brain regions in children with ASD.

The nucleus locus coeruleus (LC) is a brainstem nucleus located on the dorsal side of the pons (Figure 1), which maintains the desynchronization of brain electrical activity. LC is the main nucleus that releases norepinephrine (NE), which can improve arousal level. Locus coeruleus-norepinephrine (LC-NE) neurons project widely to the cortex, hippocampus, thalamus, cerebellum, pons and medulla. The firing frequency of LC is highest during wakefulness, decreases during slow-wave sleep, and almost stops during REM sleep (23). The excitation of LC neurons promotes wakefulness, while inhibition of LC neurons reduces wakening and promotes REM sleep (24, 25).

Clinical data showed an abnormally enhanced activity of the LC-NE system in children with ASD. Resting eye-tracking task confirmed that the resting pupil diameter of children with ASD was significantly increased, suggesting increased tonic activity of LC-NE (26). Moreover, the NE levels are increased in the cortex, cerebellum and pons of the valproic acid rat model of autism, suggesting abnormal activation of NE system in the brain (27). The hyperactivity of the LC-NE system in the brain may lead to increased arousal and reduced sleep time in children with ASD. However, conflicting data showed the blockage of norepinephrine synthetase and reduced urine norepinephrine metabolites in children with ASD, which may suggest the reduced NE level in the blood (28). This implies that the NE level changes are complicated.

LC-NE hyperactivity in ASD patients may be related to decreased inhibition of γ-aminobutyric acid (GABA). LC neuronal activity is regulated by GABAergic interneurons. Animal experiments showed that presynaptic GABA release of LC neurons in ASD model mice was reduced, leading to hyperactivity of LC neurons (29). Changes in several genes participating in the synthesis, storage, and release of GABA may lead to a decrease in GABAergic inhibition. Mutations in the *MECP2* gene, which encodes the transcriptional regulator *methyl-CpG-binding protein 2 (MECP2)*, cause autism-like stereotypies and Rett syndrome (30). *MECP2*-deficient GABAergic neurons show a reduction of presynaptic glutamic acid decarboxylase (GAD) levels, indicating a decrease in GABA synthesis (31). Experiments on Epac2−/− mice, a model of ASD, showed an alteration of *vesicular GABA transporter (VGAT)* expression, which may affect the release of GABA (32). Additionally, a recent study showed that mutations in *solute carrier family 6 member 1* (*SLC6A1*) are associated with autism. The GABA transporter1 (GAT-1) encoded by *SLC6A1* is responsible for GABA reuptake into presynaptic neurons and glial cells to regulate neurotransmission (33). Thus, mutations in the *SLC6A1* gene in certain types of ASD patients may affect the reuptake of GABA.

### 2.2. Histamine system in the posterior hypothalamus

The posterior hypothalamus is one of the brain regions involved in arousal (Figure 1), in which the excitability of histaminergic neurons can promote and maintain the state of arousal. Histaminergic neurons are active during wakefulness and inactive during NREM and REM sleep. Histamine receptor agonists induce arousal, while antagonists promote sleep (34, 35). Wright et al. compared histamine receptor-related genes in the brains of ASD patients with healthy controls, including *histidine decarboxylase* gene *(HDC)*, *histamine N-methyltransferase (HNMT), histamine receptor H1 (HRH1), histamine receptor H2 (HRH2), histamine receptor H3 (HRH3)*, *histamine receptor H4 (HRH4)*. They found that the expression of *HRH1*, *HRH2*, and *HRH3* genes in ASD patients was higher than in healthy controls (36). The changes of gene encoding histamine receptor lead to the increase of histamine receptors in sleep-related nuclei in the brain, which amplifies the effect of histamine on promoting arousal, and this may be the reason for the prolongation of the arousal period in children with ASD.

### 2.3. The Posterolateral hypothalamic orexin system

The posterolateral hypothalamus is a brain region that promotes and maintains arousal, which is rich in orexinergic neurons (Figure 1). Orexinergic neurons are necessary for maintaining waking and behavioral arousal and widely project to sleep-related brain regions, including the LC basal forebrain (BF), tuberomammillary nucleus (TMN), and dorsal raphe nuclei (DRN) (18). These projections can significantly improve the excitability of arousal-related neurons, shorten sleep duration, and maintain the arousal state. Clinical data have shown that children with ASD have higher plasma orexin levels than healthy controls (control = 6, ASD = 18) (37). The increased activity of the orexinergic system is thought to be involved in insomnia in ASD (38).

The activity of orexinergic neurons is regulated by the amygdala, and abnormal amygdala function can cause hyper excitation of orexinergic neurons. Some ASD patients have structural and functional abnormalities in the bilateral amygdala, including an increase in volume and cell density in the amygdala (39), as well as inhibition of amygdala neuron synchronization activity (38). The functional magnetic resonance imaging (fMRI) results showed that the functional connectivity between the amygdala and other brain regions was reduced (40), leading to a weakening of its regulatory role in various physiological functions (41). Some gene mutations may lead to structural and functional abnormalities of the amygdala in the brain of patients with ASD. Missense variants at R230 and R227 in the *potassium voltage-gated channel subfamily Q Member 3* (*KCNQ3*) have been reported in some autistic patients. Further patch clamp analysis in amygdala neurons showed that variants of the *KCNQ3* gene resulted in abnormal function of voltage-gated potassium channels (42), which may disturb the modulating effects of amygdala neurons on orexin neurons and prolong waking time (43). In addition, the deletion of the *protocadherin 10* (*PCDH10*) gene has also been reported in ASD patients (44). *PCDH10* is an activity-regulated gene that is expressed at high levels in olfactory and limbic regions, including the basolateral amygdala. It is implicated in social and emotional behavior phenotypes in ASD (45). Mice lacking one copy of *PCDH10* (*PCDH10+/−*) show reduced levels of N-methyl-D-aspartate receptor subunits in the amygdala (45), which wakens the regulatory effects of the amygdala on orexinergic neurons. Thus, orexinergic neurons show hyperexcitation, leading to prolonged awakenings in ASD patients.

### 2.4. Dopamine system and the Substantia Nigra pars Compacta in midbrain

The substantia nigra pars compacta and ventral tegmental areas are arousal-related nuclei, where most human dopaminergic neurons are located, and the dopamine secreted by them can effectively promote and maintain arousal (Figure 1). It was found that the increase of dopamine concentrations in substantia nigra pars compacta in midbrain significantly prolonged wakening time and shortened REM sleep. The treatment of dopamine receptor agonists significantly increased wakefulness, while antagonists augmented REM sleep (46).

Mutations of several genes participating in dopamine synthesis, catabolism and reuptake were found in ASD patients. The *Abelson-helper integration site 1 (AHI1)* gene is a candidate gene of ASD (47). Down-regulation of the expression of the rate-limiting enzyme in dopamine biosynthesis, tyrosine hydroxylase (TH), in *AHI1*-knockout (KO) mice is responsible for *AHI1*-deficiency-mediated autism symptoms. The *AHI1*-knockout autism mouse model showed low expression of TH and a decrease in dopamine synthesis (48). In addition, the deficiency of the *Rho Guanine Nucleotide Exchange Factor 10 (ARHGEF10)* gene decreased the expression of *Monoamine oxidase A (MAOA)*, one of the enzymes in the catabolism of dopamine, and increased the level of dopamine in ASD model mice (49). The *Ubiquitin protein ligase E3A (UBE3A)* gene is another candidate gene of ASD (47). The deletion of the *UBE3A* gene is the cause of the disease in certain children with ASD, which reduces dopamine transporter (DAT) function and thus affects dopamine reuptake, increasing dopamine levels and wakefulness (50). Both clinical evidence and animal model experiments have shown that certain types of syndromic autism are associated with mutations in genes encoding DAT (50–53). Apart from that, mutations in the *solute carrier family 6 member 3* (*SLC6A3*) gene also occur in clinical ASD patients (52). Mutation in the *SLC6A3* gene causes abnormal DAT function and continuous outflow of dopamine from cells, leading to an abnormal increase in extracellular dopamine concentrations (53). Therefore, in ASD patients with mutations in the *AHI1*, *UBE3A* and *SLC6A3* genes, abnormal dopamine synthesis, catabolism and reuptake result in increased dopamine concentrations in the brain, which may lead to a prolonged wakening period.

## 3. Genetic and neural mechanisms of NREM sleep abnormalities

### 3.1. Thalamic reticular nucleus

Studies showed that the functional connection between the cortex and thalamus is important for sleep regulation, which is regulated by nerves and exhibits dynamic changes (54, 55). The data from fMRI showed that the thalamocortical functional connectivity of healthy adults decreased during NREM sleep, and the information from the thalamic afferent cortex was reduced. This ensures that people enter NREM sleep from an arousal state (54). However, fMRI data showed a lack of regulation of thalamo-cortical functional connectivity in the brain of children with ASD, resulting in an abnormal increase in thalamo-cortical functional connectivity, which led to an inability to transition smoothly from wakefulness to NREM sleep (55). The thalamic reticular nucleus (TRN) is specifically excited by the thalamus cortex system and then inhibits the activity of the whole dorsal thalamus through its GABAergic neuron fiber projection, which makes TRN become the inhibitory gate to control the thalamo-cortical circuit. TRN can effectively control the thalamo-cortical functional connection and plays a sedative and sleep-promoting effect (56).

TRN is mainly composed of GABAergic neurons. The slow synchronous oscillation generated by TRN neurons can block the processing of sensory information in the cerebral cortex and limbic system, thus promoting sleep. After photogenetic activation of TRN neurons, the NREM sleep period of mice was prolonged, and the frequency and amplitude of δ waves were also increased, while muscular tension and activity in the mice decreased (57). Moreover, TRN neuronal activity is important in regulating the formation of spindle oscillations during NREM sleep. Spindle oscillation is a characteristic brainwave during the NREM sleep period, which can reduce the influence of external stimulation on the brain during sleep and stabilize sleep. Spindle oscillation is caused by the low-threshold Ca^2+^ current induced by the periodic firing of TRN cells. Therefore, spindle oscillation disappears after cutting off the connection between cortical-thalamic circuit cells and TRN neurons (58).

*Patched domain containing protein 1 (PTCHD1)* gene maps to chromosome Xp22.11 and encodes *PTCHD1*, which is closely related to TRN neuronal activity (59). During early development in mice, *PTCHD1* is selectively expressed in the TRN and continues to be highly expressed into adulthood (60). The firing activity of TRN neurons in *PTCHD1* knockout mice was reduced during sleep, leading to a reduction in NREM sleep spindle waves (60). In clinical practice, about 1% of ASD patients have *PTCHD1* gene mutations, which cause a significant reduction in gene transcription activity (61). Furthermore, the volume of TRN neurons in ASD model mice is larger than that in wild-type mice (62). These results suggest that the abnormal structure and function of TRN neurons may be responsible for the shortened sleep duration and reduced spindle wave in NREM sleep in some children with ASD.

### 3.2. Short-chain fatty acids

Short-chain fatty acids are important metabolites produced by human colonic flora, including acetate, propionate, butyrate and so on (63). Butyrate can prolong the time of NREM sleep through hepatoportal butyrate-sensitive mechanisms (64). A gut flora metabolism study found that many patients with ASD have intestinal microbiota disorders, and the level of short-chain fatty acids such as butyric acid in the brain is generally lower than that of healthy controls (65). The level of butyric acid may be associated with complex changes of butyric acid-producing bacteria. A study on butyric acid-producing bacteria showed that the abundance of butyric acid-producing bacteria Faecalibacterium and Agathobacter were significantly reduced in children with ASD who have sleep disorders (65). However, Roseburia intestinalis, another butyrate-producing bacterium, has a significantly higher abundance in children with ASD compared to control groups (66). Furthermore, it was reported that the abundance of genes related to butyric acid production decreased in the metagenome of ASD patients (67). Therefore, the complex changes of butyric acid-producing bacteria and the decreased abundance of genes associated with butyrate production may result in the decrease of butyric acid, which subsequently shortens NREM sleep.

### 3.3. Iron

Iron is important for sleep maintenance and the synthesis of neurotransmitters, such as serotonin, noradrenaline, dopamine, glutamate and γ-aminobutyric acid (GABA) (68). Iron deficiency can lead to abnormal metabolism of sleep-related neurotransmitters and worse sleep quality (69). Children with ASD had significantly lower serum ferritin levels compared to healthy controls (70). The analysis results of a retrospective chart review also showed that the serum iron level of ASD patients was lower than that of the control group (70). The above evidence suggests that iron deficiency in ASD patients can lead to abnormal metabolism of sleep-related transmitters and abnormal formation of sleep spindles, which may contribute to NREM sleep abnormalities in ASD patients.

## 4. Neural mechanisms of REM sleep abnormalities

### 4.1. Dorsal raphe nucleus

The 5-hydroxytryptamine-ergic (5-HT-ergic) neurons in the dorsal raphe nucleus (DRN) have the function of promoting wakefulness and reducing REM sleep, which are known as REM-off neurons (Figure 1). The firing frequency of DRN 5-HTergic neurons during REM sleep was significantly lower than that during wakefulness. What’s more, SEP-363856, an agonist of 5-HT, could suppress REM sleep with very large effect sizes (71).

Elevated blood 5-HT levels are a biomarker of ASD. Thirty percent of ASD patients had significantly higher blood 5-HT levels compared to healthy controls (72). However, it is reported that levels of whole blood 5-HT were lower in some ASD patients compared to healthy controls (73). Mutations of multiple 5-HT related genes, such as *5-Hydroxytryptamine Receptor 2A (HTR2A), Solute Carrier Family 6 Member 4 (SLC6A4), Monoamine Oxidase A (MAOA), Monoamine Oxidase B (MAOB), Tryptophan Hydroxylase 2 (TPH2), and Vesicular Monoamine Transporters (VMATs)*, were found in certain types of ASD patients. These mutations result in an abnormal synthesis, transport, or inactivation of 5-HT and sleep disorders in children with ASD (74–78).

In addition to 5-HT-ergic neurons, there are also a large number of GABAergic neurons in the DRN that can regulate their activity. GABAergic cells projecting to the DRN are mainly located in the hypothalamus, ventral tegmental area (VTA), and locally within the DRN (79). Studies have shown that *SH3 and multiple Ankyrin repeat domains 3 (SHANK3)* -deficient mice, a model of autism, had significantly lower levels of GABA synthesis in the hypothalamus and VTA compared to controls (80). This may lead to reduced inhibition of DRN 5-HT-ergic neurons and increased activity in the DRN of ASD patients. Therefore, it is speculated that the enhanced REM-off function resulting from increased 5-HT-ergic neuron activity in the DRN may be why some children with ASD experience shorter REM sleep duration.

### 4.2. Amygdala

An article recently published in *Science* reported that the initiation of REM sleep is modulated by the amygdala (Figure 1) (81). When the dopamine level in the amygdala was instantaneously increased through photogenetic activation, NREM sleep in mice was terminated immediately, and REM sleep began simultaneously. The study confirmed that dopamine induces a transition from NREM sleep to REM sleep by binding to dopamine type II receptors on amygdala neurons (81), while inhibiting amygdala activity results in a decrease in REM sleep duration (82).

The structure and function of the amygdala in ASD patients can undergo complex changes. Abnormal changes in dopamine concentrations in the brain of ASD patients were described in section 2.4. *Calcium-dependent secretion activator 2 (CADPS2)* gene encodes a calcium binding protein that regulates the exocytosis of synaptic secretory granules, including monoamines and neuropeptides (83). Some ASD patients have a missense variant of the *CADPS2* gene in the amygdala, which can block the release of dopamine (84, 85). Additionally, as described in section 2.3, amygdala neurons in ASD have abnormal structure and function. Therefore, the complex changes in the amygdala and the dopamine system in ASD may result in abnormal binding of dopamine to type II dopamine receptors in amygdala neurons, which could be responsible for abnormal REM sleep in some children with ASD.

## 5. Genetic and neural mechanisms of abnormal sleep–wake rhythm transition

### 5.1. Melatonin

Melatonin is an important hormone involved in the regulation of the sleep–wake cycle and the circadian rhythm, which is synthesized by the pineal gland at night (Figure 1). After binding to melatonin receptors in the suprachiasmatic nucleus, melatonin can promote sleep initiation and attenuate the arousal of the circadian clock. Studies have shown a sharp increase in nighttime sleep tendency in the hours after endogenous melatonin production (86).

Melatonin has been shown to be released at lower levels in individuals with ASD compared to healthy individuals (87). However, other research shows no significant difference in melatonin levels between 40% of ASD individuals and healthy controls (88). Melatonin treatment can increase sleep duration in ASD patients (89), suggesting that their sleep disorders are related to pineal dysfunction and lack of melatonin. Mutations in the *Acetylserotonin O-Methyltransferas (ASMT), Melatonin Receptor 1A (MTNR1A), and Melatonin Receptor 1B (MTNR1B)* genes can induce sleep disorders in ASD patients by affecting melatonin production and utilization (90–92). *ASMT* is an enzyme gene involved in melatonin synthesis, while *MTNR1A* and *MTNR1B* are melatonin receptor genes. Therefore, decreased melatonin levels due to pineal gland dysfunction may contribute to circadian rhythm sleep–wake disorders in children with ASD.

### 5.2. Basal forebrain

Acetylcholine is an important neurotransmitter associated with arousal. There are two groups of acetylcholinergic neurons in the human body: one is in the brainstem, while the other is in the basal forebrain (BF) (Figure 1). Cholinergic neurons in the basal forebrain play a role in cortical activation during the sleep–wake cycle, promoting the transition from NREM sleep to the waking state or to REM sleep. The level of acetylcholine in the basal forebrain is high during the arousal period and REM sleep, and low during slow wave-sleep (93).

Neuroanatomical analysis revealed that autistic patients show smaller neurons and increased cell density in the BF as compared to age-matched healthy individuals (94). Moreover, diminished activity of cholinergic neurons in the basal forebrain was reported in mice (95). Therefore, BF lesions in children with ASD may cause abnormal function of BF cholinergic neurons, resulting in their abnormal regulation of phase conversion during sleep. This may be one of the neural mechanisms causing circadian rhythm sleep–wake disorders in children with ASD.

## 6. Limitations

In the present review, we limited our search to articles published in English. Thus, we may have missed related articles published in other languages. In addition, terminology related to genetic and neurological abnormalities varies, which may result in missing related articles due to variability in keyword use. Moreover, we did not restrict our review to studies with human subjects. Therefore, further investigation in ASD patients is required to replicate some of the reported findings in animal models of ASD. Finally, we did not consider sex differences in sleep disorders in children with ASD.

## 7. Conclusion

**Figure 2:**
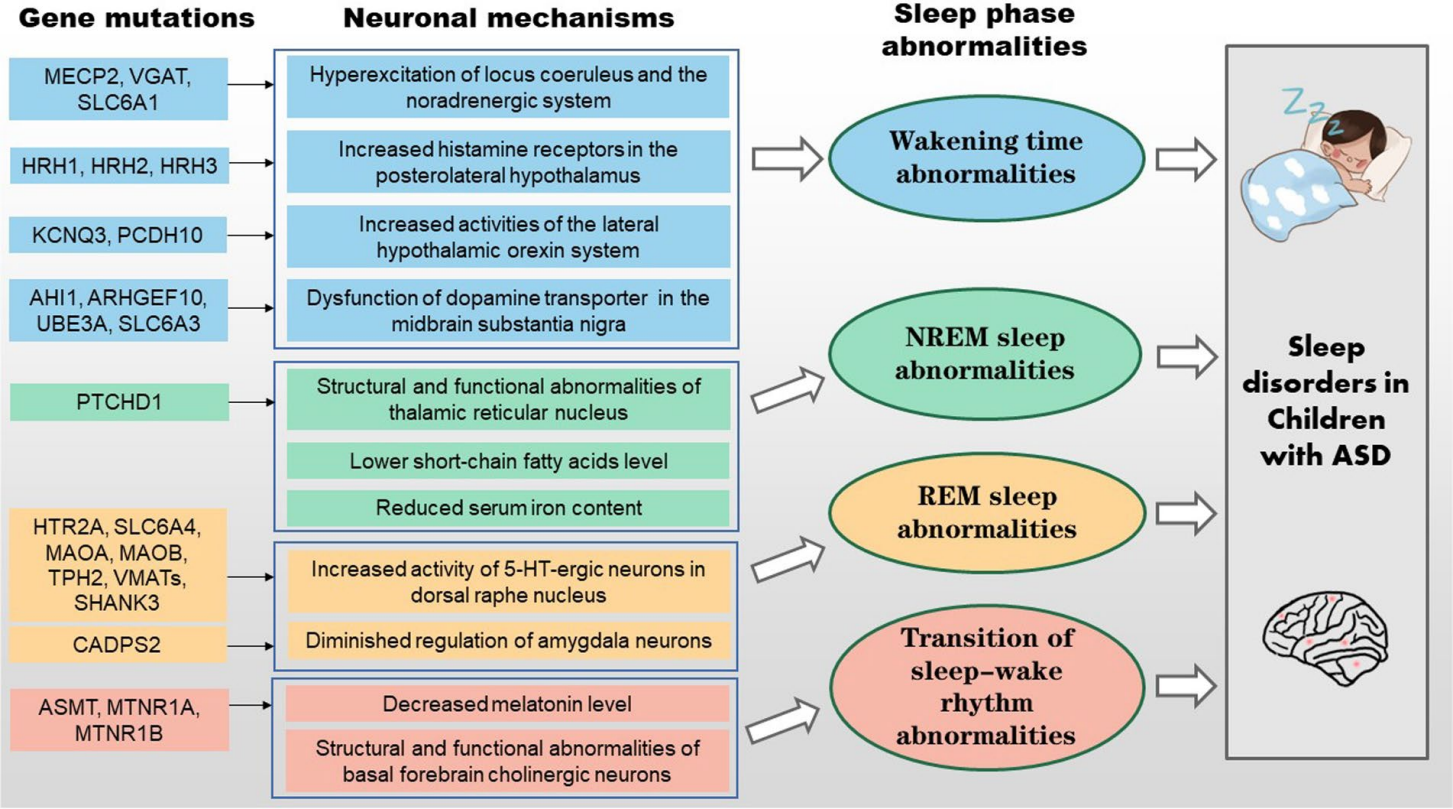
Schematic representation of the genetic and neural mechanisms of sleep disorders in children with ASD.

There is an established relationship between genetic mutations/neurological abnormalities and sleep disorders in children with ASD. As shown in Figures 1, 2, mutations in the *MECP2*, *VGAT, SLC6A1*, *SLC6A3*, *HRH1-3*, *KCNQ3*, *PCDH10*, *AHI1*, *UBE3A* and *ARHGEF10* genes can cause atypical activity of wake-related neural circuits, which may lead to sleep problems such as prolonged sleep latency, short total sleep duration, and waking up at night. Additionally, butyric acid, iron deficiency, and TRN dysfunction are also linked to NREM sleep disorder in children with ASD. Mutations in the *HTR2A*, *SLC6A4*, *MAOA*, *MAOB*, *TPH2, VMATs, SHANK3,* and *CADPS2* genes induce abnormal reactions of REM-off and REM-on neurons, which may lead to NREM disorder. Mutations in the *ASMT*, *MTNR1A* and *MTNR1B* genes induce the decreased synthesis and secretion of melatonin, which may cause abnormal sleep–wake rhythm transition. However, more research is needed on the clinical intervention of sleep disorders in children with ASD, as there are few intervention studies available. Overall, the genetic and neural mechanisms of sleep disorders in children with ASD warrant further investigation.

## Author contributions

QJ, S-JL, J-BZ, YX, X-HD, C-XW, L-ML, J-YT and Z-RZ contributed to the writing and editing of this review. X-HD and Z-RZ conceived the scope of the review, coordinated efforts among authors, and wrote the bulk of the wake sections. QJ, S-JL, J-BZ and YX wrote the bulk of the sleep section. QJ and LS-J wrote the bulk of the sleep–wake rhythm section. All authors contributed to the article and approved the submitted version.

## Funding

This work was supported by grants from the National Natural Science Foundation of China (31671106), the Scientific Foundation of Chongqing (Cstc2019jcyj-msxmX0019), and the Science Foundation of Army Medical University (2019XYY08).

## Conflict of interest

The authors declare that the research was conducted in the absence of any commercial or financial relationships that could be construed as a potential conflict of interest.

## Publisher’s note

All claims expressed in this article are solely those of the authors and do not necessarily represent those of their affiliated organizations, or those of the publisher, the editors and the reviewers. Any product that may be evaluated in this article, or claim that may be made by its manufacturer, is not guaranteed or endorsed by the publisher.
